# Supplementary material for: Anabolic Peptide-Enriched Stealth Nanoliposomes for Effective Anti-Osteoporotic Therapy
Source: Pharmaceutics. 2022 Nov 9;14(11):2417. doi: 10.3390/pharmaceutics14112417 (PMC9697760; doi:10.3390/pharmaceutics14112417)
Supplement: Supplementary file 1 [file pharmaceutics-14-02417-s001.zip › pharmaceutics-1998370-supplementary.pdf]

Supplementary Material

# Anabolic Peptide Enriched Stealth Nanoliposomes for Effective Anti-osteoporotic Therapy

**Sagar Salave, Dhwani Rana, Hemant Kumar, Nagavendra kommineni, and Derajram Benival**

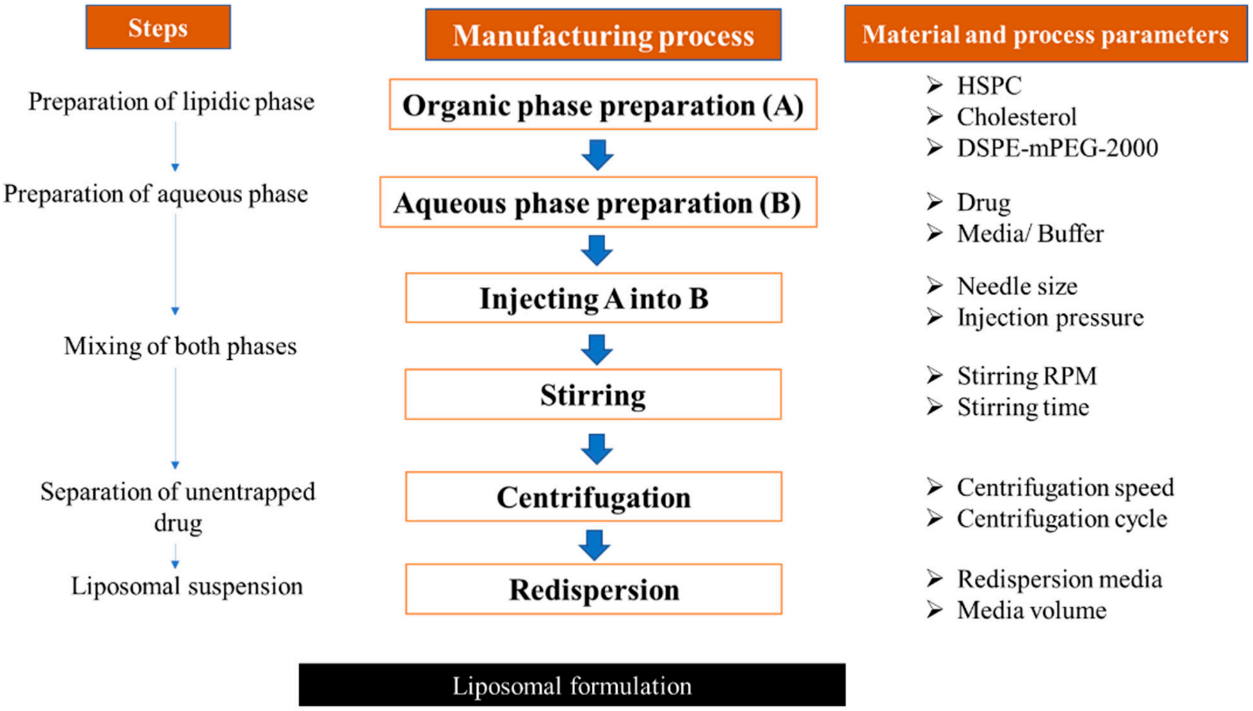


**Figure S1**. Process map for formulation development of liposomal product using ethanol injection method.

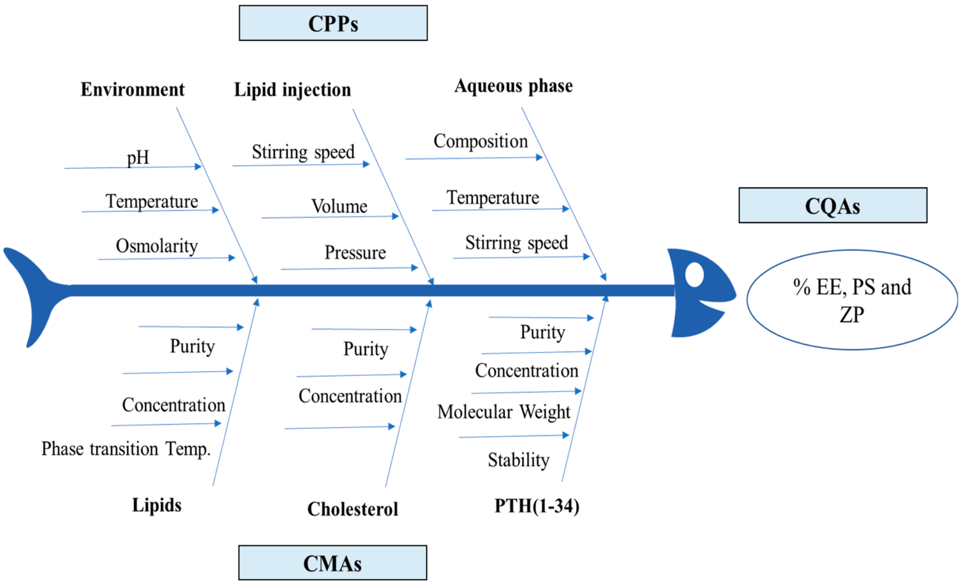


**Figure S2.** Ishikawa fishbone diagram.

**Table S1**. Layout of experimental design

| Run no | lipid concentration (mM) | cholesterol concentration (mM) | stirring rate (rpm) | particle size (nm) | EE (%) |
|---|---|---|---|---|---|
| 1 | 30 | 10 | 400 | 92.31 | 84.57 |
| 2 | 30 | 15 | 500 | 155.83 | 76.96 |
| 3 | 30 | 10 | 600 | 97.51 | 62.02 |
| 4 | 40 | 10 | 500 | 94.33 | 87.98 |
| 5 | 40 | 15 | 400 | 132.17 | 53.64 |
| 6 | 30 | 15 | 500 | 120.70 | 47.48 |
| 7 | 20 | 15 | 400 | 39.48 | 80.29 |
| 8 | 30 | 15 | 500 | 104.03 | 53.92 |
| 9 | 30 | 15 | 500 | 76.58 | 39.16 |
| 10 | 30 | 15 | 500 | 71.02 | 24.58 |
| 11 | 20 | 20 | 500 | 54.19 | 40.20 |
| 12 | 20 | 10 | 500 | 77.41 | 46.18 |
| 13 | 20 | 15 | 600 | 97.81 | 60.31 |
| 14 | 30 | 20 | 600 | 65.00 | 48.00 |
| 15 | 30 | 20 | 400 | 77.65 | 32.99 |
| 16 | 40 | 20 | 500 | 95.21 | 58.16 |

| | | | | | |
|---|---|---|---|---|---|
| 17 | 40 | 15 | 600 | 141.93 | 60.44 |

**Table S2**. Statistical data of model terms

| Terms | Particle size (nm) | EE (%) | Inference |
|---|---|---|---|
| **Model p-value** | 0.0142 | 0.0432 | Significant |
| **Model F value** | 5.86 | 4.88 | |
| **Lack of Fit** | 0.9486 | 0.7761 | Non- significant |
| **$R^2$** | 0.4556 | 0.2453 | |
| **Adjusted $R^2$** | 0.3778 | 0.1950 | |
| **Predicted $R^2$** | 0.2234 | 0.0395 | |
| **Adeq Precision** | 6.9909 | 4.5523 | |

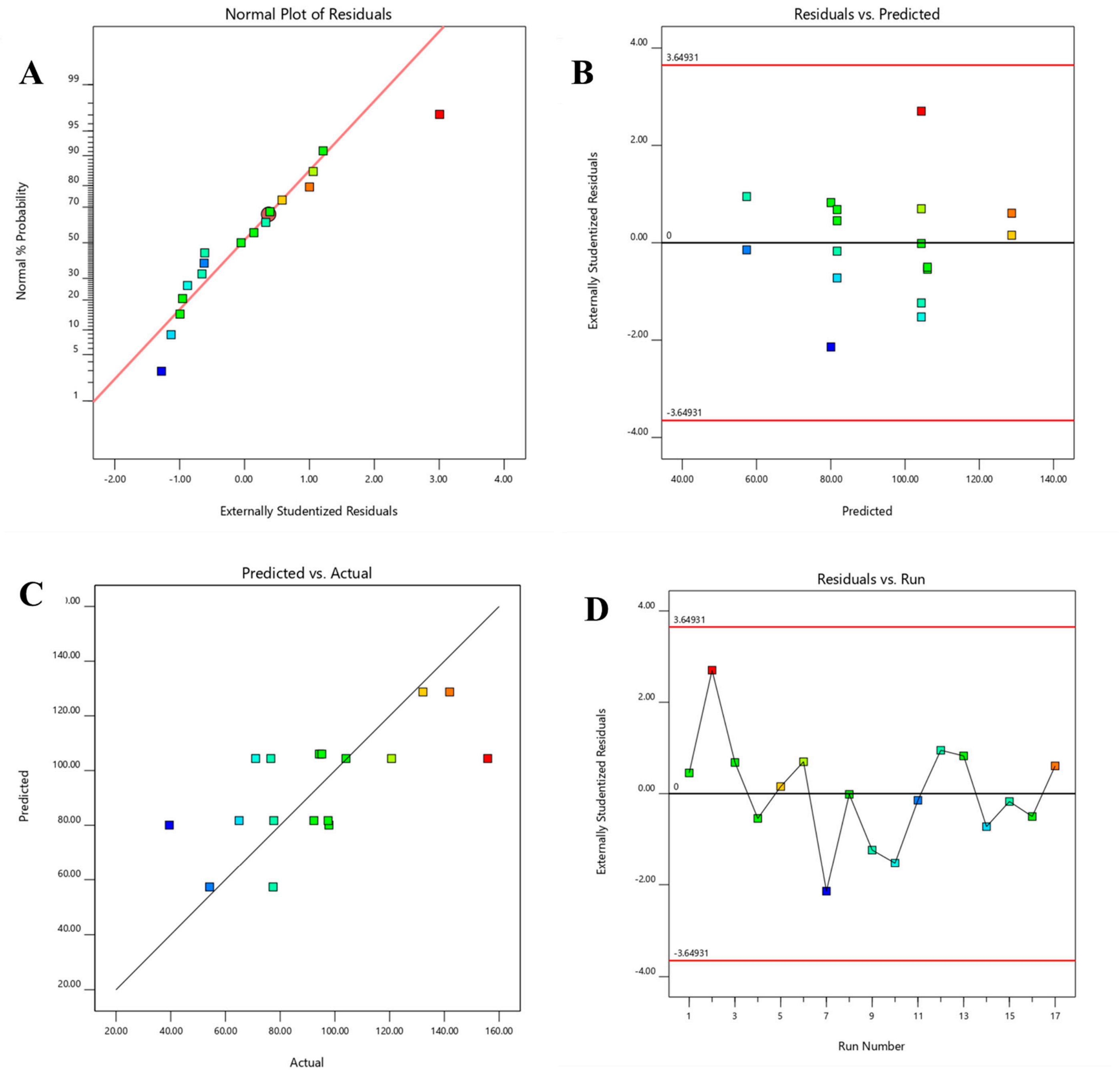


**Figure S3**. Residual analysis for particle size. (A) Normal plot of residuals, (B) Residual versus Predicted plot, (C) Predicted versus actual plot, (D) Residual versus run plot.

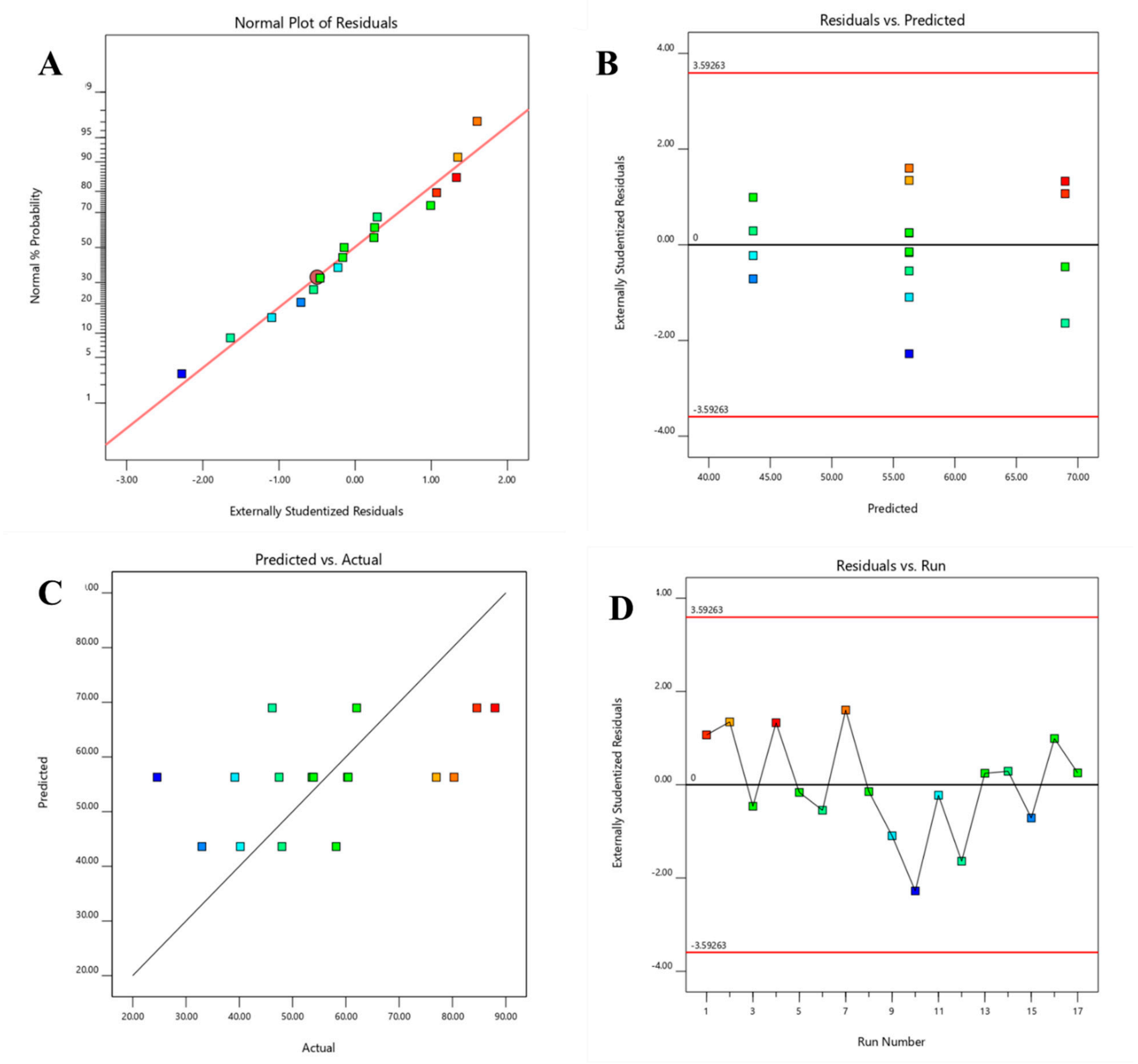


**Figure S4**. Residual analysis for %EE. (A) Normal plot of residuals, (B) Residual versus Predicted plot, (C) Predicted versus actual plot, (D) Residual versus run plot.

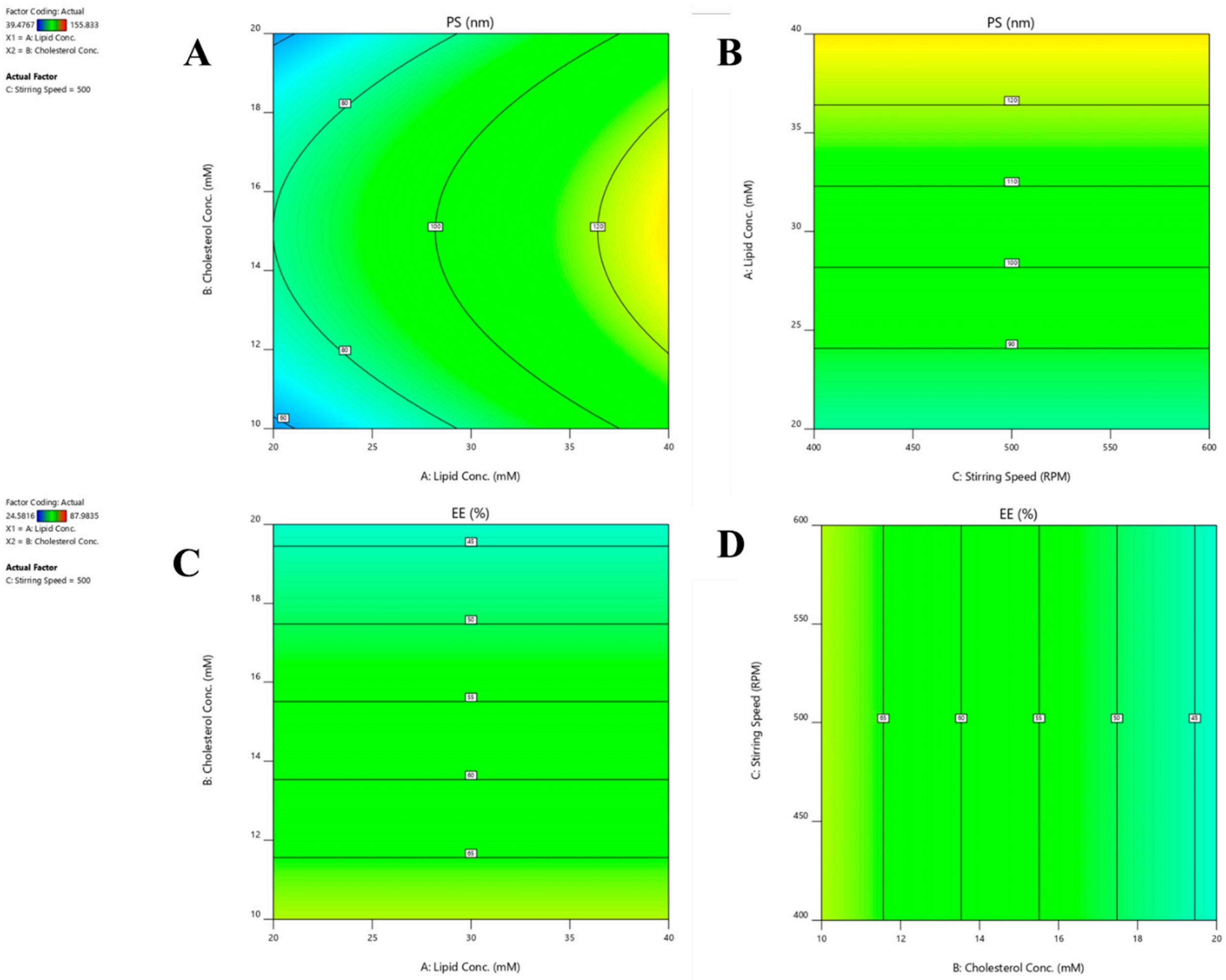


**Figure S5**. Counter plot. (A-B) for the particle size, (C-D) for %EE.

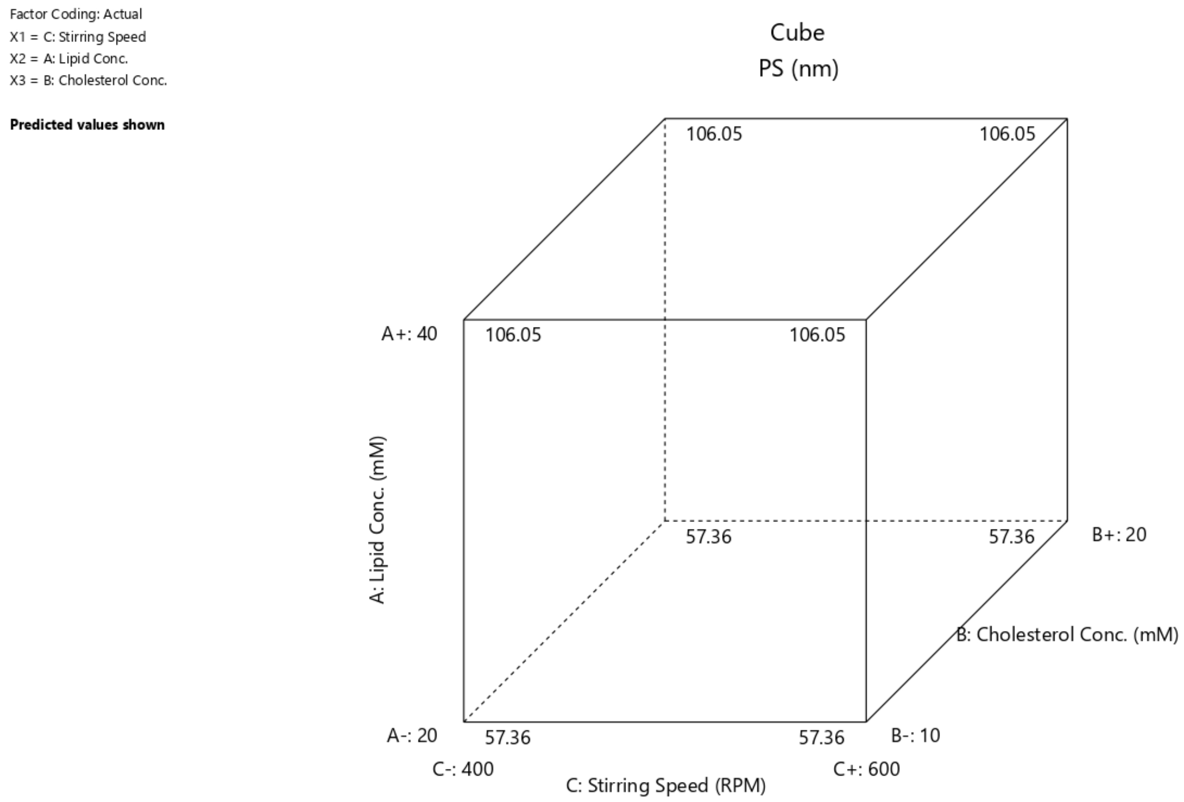


**Figure S6**. Box plot for particle size.

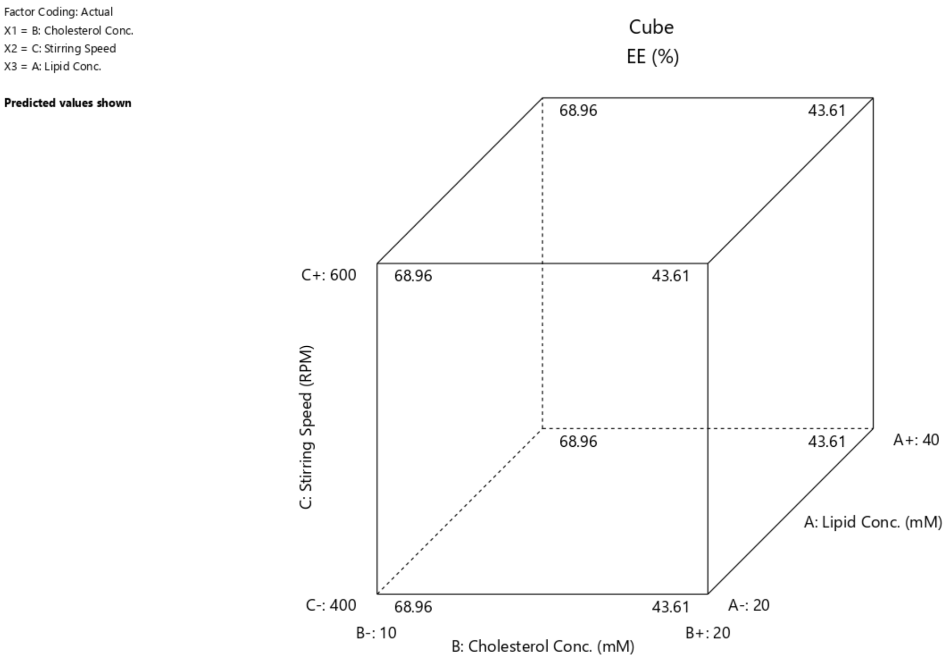


**Figure S7.** Box plot for %EE.

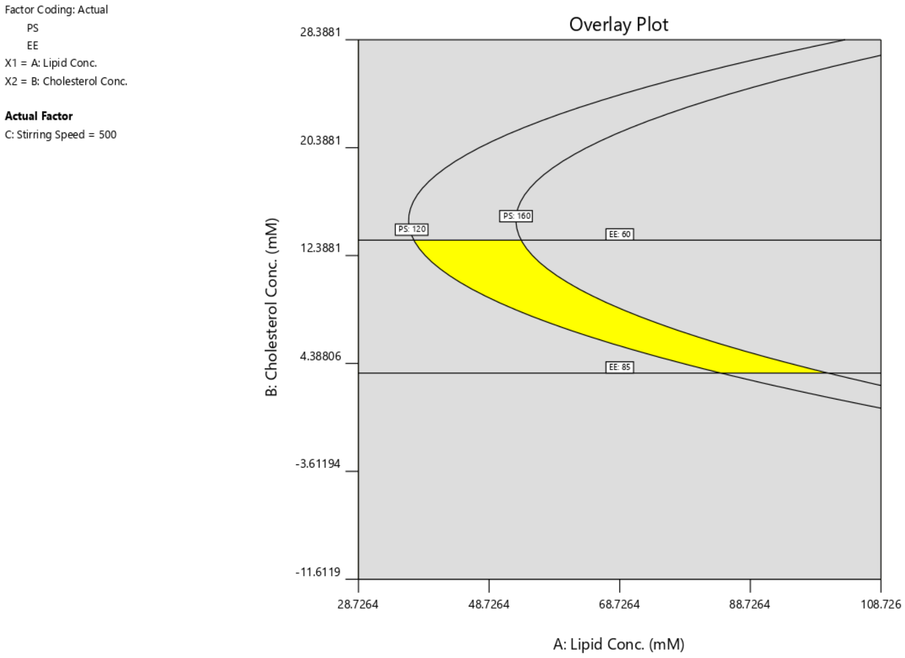


**Figure S8**. Design space for PTH-LPs
